# Preventive effect of Ibrolipim on suppressing lipid accumulation and increasing lipoprotein lipase in the kidneys of diet-induced diabetic minipigs

**DOI:** 10.1186/1476-511X-10-117

**Published:** 2011-07-16

**Authors:** Yi Liu, Zong Bao Wang, Wei Dong Yin, Qin Kai Li, Man Bo Cai, Jian Yu, Hong Guang Li, Chi Zhang, Xiu Hong Zu

**Affiliations:** 1 Department of Laboratory Animal Science, School of Pharmacy and Life Science, University of South China, Hengyang, Hunan 421001, China; 2 Department of Biochemistry, Medical College, Shaoguan University, Shaoguan, Guangdong 512026, China; 3 Key Laboratory for Atherosclerology of Hunan Province, Institute of Cardiovascular Research, University of South China, Hengyang, Hunan 421001, China

**Keywords:** Lipoprotein lipase, Lipoprotein lipase activator, Lipid accumulation, Diabetic nephropathy, Swine, Miniature

## Abstract

**Background:**

The role of renal lipoprotein lipase (LPL) *per se *in kidney diseases is still controversial and obscure. The purpose of this study was to observe the preventive effects of Ibrolipim, a LPL activator, on lipid accumulation and LPL expression in the kidneys of minipigs fed a high-sucrose and high-fat diet (HSFD).

**Methods:**

Male Chinese Bama minipigs were fed a control diet or HSFD with or without 0.1 g/kg/day Ibrolipim for 5 months. Body weight, plasma glucose, insulin, lipids, LPL activity, and urinary microalbumin were measured. Renal tissue was obtained for detecting LPL activity and contents of triglyceride and cholesterol, observing the renal lipid accumulation by Oil Red O staining, and examining the mRNA and protein expression of LPL by real time PCR, Western Blot and immunohistochemistry.

**Results:**

Feeding HSFD to minipigs caused weight gain, hyperglycemia, hyperinsulinemia, hyperlipidemia and microalbuminuria. HSFD increased plasma LPL activity while it decreased the mRNA and protein expression and activity of LPL in the kidney. The increases in renal triglyceride and cholesterol contents were associated with the decrease in renal LPL activity of HSFD-fed minipigs. In contrast, supplementing Ibrolipim into HSFD lowered body weight, plasma glucose, insulin, triglyceride and urinary albumin concentrations while it increased plasma total cholesterol and HDL-C. Ibrolipim suppressed the renal accumulation of triglyceride and cholesterol, and stimulated the diet-induced down-regulation of LPL expression and activity in the kidney.

**Conclusions:**

Ibrolipim exerts renoprotective and hypolipidemic effects *via *the increase in renal LPL activity and expression, and thus the increased expression and activity of renal LPL play a vital role in suppressing renal lipid accumulation and ameliorating proteinuria in diet-induced diabetic minipigs.

## Introduction

It is now increasingly accepted that abnormal lipid metabolism and renal accumulation of lipids play an important role in the pathogenesis of diabetic nephropathy (DN) [1-3]. Numerous experimental studies have shown the accumulation of triglyceride and cholesterol in the kidney of animal models fed by a diet rich in cholesterol and (or) saturated fat and the role of dyslipidemia in promoting kidney damage [4-6]. Our recent works have suggested that feeding high-fat/high-sucrose/high-cholesterol diet to minipigs induces insulin resistance [7,8], moderate glomerulosclerosis and early-stage DN [8]. Understanding the mechanisms whereby lipids initiate and augment DN is an important unresolved question.

Lipoprotein lipase (EC 3.1.1.34; LPL) plays a central role in lipid metabolism and transports by catalyzing the hydrolysis of the triglyceride (TG) component of circulating chylomicrons (CM) and very-low-density lipoproteins (VLDL) to provide non-esterified fatty acids and 2-monoacylglycerol for tissue utilization [9]. Considering tissue-specific regulation and effects of LPL in lipid metabolism [10], the question arises whether LPL is involved in the mechanism of DN. LPL is expressed in mesangial and/or tubular epithelial cells, and localizes to the endothelial cells as in the blood vessels [11,12]. LPL enhances VLDL binding to glomerular mesangial cells and induces proliferation and platelet-derived growth factor (PDGF) expression independent of LPL's familiar role in triglyceride hydrolysis [13]. Contradictorily, Li et al [14] find that VLDL-induced triglyceride accumulation in human mesangial cells is mainly mediated by the enzymolysis action of LPL. On the contrary, there are many reports that LPL expression and activity in the heart, skeletal muscle, and adipose tissues are suppressed in humans and experimental animals with kidney disease [15-18]. In any case, these conflicting results suggest that LPL may be important in mediating the initiation and/or progression of renal disease. In view of the strong connection between LPL and kidney disease, however, it is surprising that there are few direct studies on the role of renal LPL. It is unknown, up to now, whether changing the expression of LPL in kidney *per se *influences renal lipid metabolism and contributes to the progression of DN *in vivo*.

Ibrolipim (NO-1886) is a novel compound that has been reported to increase LPL activity and mass in postheparin plasma and LPL mRNA and activity in adipose tissue, myocardium and skeletal muscle, resulting in a reduction of plasma TG with concomitant elevation of high-density lipoprotein cholesterol (HDL-C) in rats [19-21]. Furthermore, Ibrolipim decreases visceral and subcutaneous fat accumulation and ectopic lipid deposition in the heart, skeletal muscle, liver and pancreas in diet-induced diabetic animal models [7,22,23]. In rat model of nephrotic syndrome induced by adriamycin and a high-protein diet, Ibrolipim reduces plasma creatinine (Cr), blood urea nitrogen and proteinuria, and ameliorates tubulointerstitial lesions [24]. However, the mechanisms responsible for the renoprotective effect of Ibrolipim in nephrosis are not fully understood. Moreover, whether Ibrolipim affects the expression of LPL and lipid accumulation in kidney is still unknown.

The purposes of the present study, therefore, are to examine (a) whether there is LPL expression in the kidney of Chinese Bama minipigs, (b) whether the high-sucrose and high-fat diet causes renal lipid accumulation and influences renal LPL expression, (c) whether administration of Ibrolipim can suppress diet-induced lipid accumulation and improve renal injury through changing renal LPL expression, and (d) whether LPL plays an important role in the pathogenesis of DN.

## Materials and methods

### Materials

Ibrolipim (NO-1886), 4-diethoxyphosphorylmethyl-*N*-(4-bromo-2-cyanophenyl) benzamide (CAS 133208-93-2), in powder form was synthesized in the New Drug Research Laboratory of Otsuka Pharmaceutical Factory Inc. (Tokushima, Japan). Antibody against LPL was purchased from Boster Biotech, Inc. (Wuhan, China). Immunohistochemical kits were used from the EliVision™ plus HRP System (Maixin Biotech Inc., Fuzhou, China). All other chemicals used were high-grade commercially available products.

### Animals

Fifteen male Chinese Bama miniature pigs, 3 to 4 months, were obtained from the Laboratory Animal Center of the Third Military Medical University (Chongqing, China). They were housed in single pens under controlled conditions (temperature of 20 ± 2°C and relative humidity between 30% to 70%) and randomized into three groups with similar body weight: n = 5 in the normal control diet (CD) group; n = 5 in the high sucrose and fat diet (HSFD) group; n = 5 in the HSFD supplemented with 0.1 g/kg body weight/day Ibrolipim (HSFD+Ibrolipim) group. The HSFD used in this study was 51% normal swine diet supplemented with 37% sucrose, 10% lard, and 2% cholesterol [8]. The amount of daily fodder was 4% weight of minipigs which were fed three times a day. Water was available *ad libitum*. Body weights were recorded every month, and the study period was 5 months. All experiments were performed according to the guidelines of the Animal Ethics Committee of the University of South China (No. 2005-016).

### Biochemical analysis of plasma and urine

Fasting blood samples were collected from the orbital sinus for plasma parameters at the end of each month. Glucose, TG, total cholesterol (TC) and HDL-C were measured by commercial enzymatic method kits (Rongsheng Biotech Inc., Shanghai, China). Insulin was assayed by a radioimmunoassay kit (China Institute of Atomic Research, Beijing, China). Homeostatic model assessment (HOMA) as the estimate of insulin resistance was calculated by the formula: insulin × glucose/22.5 [25].

An hour after food consumption at the end of 5^th ^month, heparin (150 U/kg) was injected into a vein in one ear, and 10 min later blood was collected from a vein in the other ear. The plasma was measured for LPL activity by an immunochemical method described previously [26] using glycerol tri[l-^14^C]oleate as substrate and selective blocking of hepatic lipase activity with antiserum to hepatic lipase.

Randomly obtained urine samples in the morning were used for determination of microalbumin (mALB) and Cr at 0, 2, 4 and 5 months. Urinary mALB was measured by immunoturbidimetry (Mingdian Biotech Inc., Shanghai, China). Plasma and urinary Cr were measured by using the Jaffe method (Jianchen Biotech Inc., Nanjing, China). The urinary mALB to Cr ratio was calculated [27].

### Biochemical analysis in renal tissue

At the end of the experimental period, the animals were killed by exsanguination under sodium pentobarbital anaesthesia. Left kidneys were removed and weighed, and calculated the kidney weight index (left kidney weight/body weight ratio, g/kg).

Total lipids were extracted from 1 g of right kidneys by the method of Bligh and Dyer [28]. Tissue TC and TG were analyzed by using the enzymatic method with commercial kits (Rongsheng), and the protein concentration was measured by the BCA Assay Kit (Pierce, Rockford, IL, USA).

Renal heparin-triggered LPL activity was measured as reported previously [19,26]. A specimen of kidney was homogenised in 50 mmol/l NH_4_Cl buffer (pH 8.5) and incubated with buffer containing heparin for 60 min at 0°C. The suspension was then centrifuged, and the supernatant was used to measure LPL activity as described above.

### Quantitative real-time PCR

Total RNA was isolated from right kidneys by TriZol (Invitrogen, Carlsbad, CA, USA) method. Then, cDNA was synthesized with Invitrogen SuperScrip preamplification system. To investigate the expression of LPL mRNA, real-time PCR (Rotor-Gene 3000 real-time analyzer, Corbett Research, Mortlake, Australia) was performed with SYBR Green JumpStart Taq ReadyMix kit (Sigma-Aldrich, St. Louis, MO, USA). The porcine gene specific sequence of LPL primer was forward 5'-CGA AGT ATT GGC ATC CAG AAA C-3' and reverse 5'-TTG ATC TCA TAG CCC AAG TTG TT-3'. The relative amount of LPL mRNA was normalized to the expression of internal control β-actin in each sample. The sequence of β-actin primer was forward 5'-CCT GTA CGC CAA CAC AGT GC-3' and reverse 5'-ATA CTC CTG CTT GCT GAT CC-3'. All the data were calculated from triplicate reactions.

### Western blotting

Proteins were extracted from left kidneys by using Protein Extraction Kit (G-Biosciences, St Louis, MO, USA). After centrifugation, supernatant was obtained to determine the protein concentration by BCA method (Pierce). Protein samples (10 μg) were subjected to SDS-PAGE (10% w/v) and then transferred to nitrocellulose membranes. Membranes were blocked in 5% dried milk in Tris-buffered saline with Tween, incubated with anti-LPL (1:400 dilution), followed by horseradish peroxidase-labeled anti-rabbit IgG (1:1000 dilution), and then developed by using the enhanced chemiluminescence detection kit (Amersham Biosciences, Piscataway, NJ, USA) and XAR sensitive film (Kodak, Rochester, NY, USA). The signals were quantified by using the Fluor-S MultiImager system (Bio-Rad Laboratories, Hercules, CA, USA). The LPL protein level was assessed by densitometry with β-actin (Sigma-Aldrich) as a control.

### HE staining and Oil Red O staining

Paraffin sections were stained for hematoxylin and eosin (HE). Frozen sections were used for Oil Red O staining to determine the renal accumulation of neutral lipid. The area of lipid droplets, appeared as red spots, was captured in more than 5 randomly chosen digital photographs (× 200) made with the Olympus microscope system (Tokyo, Japan), and quantitatively measured using Image-Pro Plus 6.0 software (Media Cybernetics, Bethesda, MD, USA), and then calculated as a percentage of the field area in each section. The mean area for each minipig in each group was calculated and compared in a blinded manner by the renal pathologists.

### Immunohistochemistry

Kidney sections were deparaffinized, hydrated and then preincubated with 0.1% trypsin for 30 min at room temperature. After quenching the endogenous peroxidase by immersion in 3% hydrogen peroxide for 10 min, sections were incubated at 4°C overnight with polyclonal antibodies against LPL diluted to 1:100, then subsequently incubated with polymerized HRP-labeled goat anti-rabbit IgG for 1 h at 4°C. The antigen was visualized with diaminobenzidine and counterstained with hematoxylin. Negative controls were included with substitution of the primary antibodies with 10% non-immune goat serum.

### Statistical analysis

Results are expressed as mean ± SD by using the SPSS version 17 software. The mALB/Cr ratio was log-transformed to approximate a normal distribution. Comparisons among the three groups were analyzed for statistical significance by using one-way analysis of variance, followed by LSD-*t *test for multiple comparisons. Statistical analysis for histological study was performed by using a nonparametric Mann-Whitney test. Correlation analysis was performed using Pearson's test. *P *values less than 0.05 were considered significant.

## Results

### Effects of Ibrolipim on body and kidney weights in minipigs

The body weights of three groups were linearly elevated with feeding duration. Body weight gain was significantly higher in the HSFD group than in the CD group, whereas it was suppressed in the HSFD+Ibrolipim group compared with the HSFD group in the 4th and 5th months (Table 1). There was no significant difference in the left kidney weight index among the three groups (CD, 3.42 ± 0.51 g/kg; HSFD, 2.89 ± 0.71 g/kg and HSFD+Ibrolipim, 3.58 ± 0.47 g/kg, *P *= 0.199).

**Table 1:** Body weight, fasting plasma glucose and lipid metabolites in Chinese Bama minipigs

Parameters	Baseline	1 month	2 months	3 months	4 months	5 months
**Body weight (kg)**						
CD	6.04 ± 1.24	9.22 ± 1.29	10.28 ± 1.51	12.34 ± 1.73	14.35 ± 2.14	16.85 ± 3.74
HSFD	6.04 ± 2.12	8.90 ± 2.68	10.76 ± 2.61	14.18 ± 4.25	18.50 ± 5.31 *	23.55 ± 7.46 **
HSFD+Ibrolipim	6.02 ± 1.10	8.48 ± 1.65	10.08 ± 1.84	13.02 ± 2.50	14.40 ± 2.51 ^#^	16.56 ± 3.83 ^##^
**Glucose (mmol/l)**						
CD	4.71 ± 0.39	4.24 ± 0.90	4.83 ± 0.87	5.24 ± 1.00	4.65 ± 0.71	4.77 ± 0.57
HSFD	5.12 ± 0.84	5.97 ± 0.73 *	6.88 ± 0.57 *	8.89 ± 2.05 **	9.54 ± 2.12 **	10.27 ± 2.25 **
HSFD+Ibrolipim	4.78 ± 0.62	4.41 ± 1.45	4.96 ± 1.09 ^#^	5.89 ± 0.50 ^#^	4.85 ± 1.10 ^##^	4.75 ± 0.45 ^##^
**Insulin (U/l)**						
CD	6.97 ± 0.33	7.31 ± 0.94	7.50 ± 0.93	8.68 ± 0.82	7.25 ± 0.80	7.65 ± 1.33
HSFD	7.47 ± 1.16	9.12 ± 1.63	15.12 ± 3.22 *	21.16 ± 5.08 *	24.43 ± 3.55 **	17.43 ± 3.80 **
HSFD+Ibrolipim	7.65 ± 0.91	8.73 ± 1.52	9.43 ± 0.84 ^#^	10.95 ± 2.95 ^#^	10.30 ± 3.57 ^##^	10.34 ± 2.71 ^#^
**Homeostatic model assessment**						
CD	1.38 ± 0.18	1.54 ± 0.50	1.63 ± 0.47	1.97 ± 0.63	1.51 ± 0.32	1.64 ± 0.45
HSFD	1.59 ± 0.26	2.29 ± 0.39	4.38 ± 1.05 **	7.04 ± 1.05 **	10.40 ± 1.75 **	7.83 ± 1.63 **
HSFD+Ibrolipim	1.56 ± 0.26	1.87 ± 0.56	2.11 ± 0.59 ^##^	2.89 ± 0.51 ^##^	2.16 ± 0.45 ^##^	2.62 ± 0.70 ^##^
**Triglyceride (mmol/l)**						
CD	0.59 ± 0.04	0.59 ± 0.06	0.63 ± 0.10	0.69 ± 0.16	0.71 ± 0.15	0.73 ± 0.13
HSFD	0.59 ± 0.05	0.94 ± 0.07 **	1.50 ± 0.18 **	1.87 ± 0.14 **	2.03 ± 0.42 **	2.10 ± 0.32 **
HSFD+Ibrolipim	0.58 ± 0.04	0.60 ± 0.01 ^#^	0.78 ± 0.08 ^##^	0.87 ± 0.17 ^##^	1.03 ± 0.25 ^##^	1.10 ± 0.35 ^##^
**Total cholesterol (mmol/l)**						
CD	2.06 ± 0.34	1.99 ± 0.33	2.01 ± 0.35	1.68 ± 0.21	1.82 ± 0.49	2.07 ± 0.46
HSFD	2.31 ± 0.44	6.24 ± 1.77 **	9.06 ± 1.44 **	11.20 ± 2.02 **	15.23 ± 2.00 **	20.45 ± 2.83 **
HSFD+Ibrolipim	2.29 ± 0.55	8.02 ± 2.15 ^#^	11.99 ± 2.15 ^#^	13.55 ± 3.12 ^#^	16.39 ± 4.23	22.14 ± 4.59
**HDL cholesterol (mmol/l)**						
CD	0.78 ± 0.14	0.76 ± 0.18	0.85 ± 0.17	1.02 ± 0.12	0.81 ± 0.16	0.96 ± 0.29
HSFD	0.89 ± 0.12	1.09 ± 0.18 *	1.37 ± 0.28 *	1.82 ± 0.53 *	2.00 ± 0.39 **	1.83 ± 0.36 **
HSFD+Ibrolipim	0.79 ± 0.15	1.23 ± 0.21	2.29 ± 0.43 ^##^	2.85 ± 0.47 ^##^	2.92 ± 0.54 ^##^	2.86 ± 0.46 ^##^

Values are means ± SD, n = 5 in each group; * *P *< 0.05, ** *P *< 0.01 *vs*. CD group; # *P *< 0.05, ## *P *< 0.01 *vs*. HSFD group.

### Effects of Ibrolipim on plasma parameters in minipigs

As shown in Table 1, plasma glucose, insulin, TG, TC and HDL-C concentrations were significantly increased in the HSFD group over the control group with feeding program. Treatment with Ibrolipim lowered plasma glucose, insulin and TG concentrations to be below those in the HSFD group. However, administration of Ibrolipim in the HSFD caused an increase of plasma TC and HDL-C, even higher than in the HSFD group. The degree of insulin resistance calculated by HOMA was high in HSFD-fed minipigs after 2 months, and suppressed in the HSFD+Ibrolipim group compared with the HSFD group.

### Effects of Ibrolipim on renal function in minipigs

As shown in Table 2, the urinary mALB/Cr ratio was significantly increased from the 4th month of HSFD-feeding compared with that of CD-feeding. Ibrolipim supplementation to HSFD markedly decreased the ratio compared with HSFD-feeding. No significant changes were observed in Cr levels of plasma and urine among three groups.

**Table 2:** Renal function in Chinese Bama minipigs

Parameters	Baseline	2 months	4 months	5 months
**Urinary microalbumin/creatinine ratio (mg/g)**				
CD	12.26 ± 2.54	12.77 ± 3.33	13.52 ± 4.67	12.60 ± 3.27
HSFD	12.73 ± 3.14	14.16 ± 3.53	25.13 ± 4.65 *	48.08 ± 11.77 **
HSFD+Ibrolipim	13.18 ± 3.45	12.66 ± 4.01	17.24 ± 5.63 ^#^	24.14 ± 7.99 ^##^
**Plasma creatinine (μmol/l)**				
CD	28.56 ± 5.54	28.97 ± 7.44	28.81 ± 6.02	29.69 ± 3.81
HSFD	26.67 ± 6.53	27.71 ± 6.36	30.76 ± 8.91	33.38 ± 9.10
HSFD+Ibrolipim	28.77 ± 7.34	30.25 ± 6.17	31.56 ± 7.43	32.19 ± 8.01
**Urinary creatinine (mmol/l)**				
CD	3.77 ± 0.53	4.08 ± 0.84	4.13 ± 1.00	4.43 ± 0.87
HSFD	4.05 ± 0.72	4.20 ± 0.92	4.08 ± 0.76	4.71 ± 0.69
HSFD+Ibrolipim	3.92 ± 0.68	4.17 ± 0.96	4.20 ± 1.01	4.48 ± 0.49

Values are means ± SD, n = 5 in each group; * *P *< 0.05, ** *P *< 0.01 *vs*. CD group; # *P *< 0.05, ## *P *< 0.01 *vs*. HSFD group.

### Effects of Ibrolipim on renal lipid accumulation in minipigs

The feeding of HSFD increased the contents of TC and TG in the renal tissues, and these increases were suppressed by the treatment with Ibrolipim (Table 3).

**Table 3:** Renal lipid contents, and renal and plasma LPL activities in Chinese Bama minipigs

Groups	Renal triglyceride content (mg/g protein)	Renal cholesterol content (mg/g protein)	Renal lipoprotein lipase activity (U/g protein)	Plasma lipoprotein lipase activity (U/ml)
CD	7.17 ± 1.53	7.49 ± 1.92	0.07 ± 0.01	7.34 ± 1.72
HSFD	15.99 ± 2.59 **	18.69 ± 3.99 **	0.05 ± 0.01 *	31.88 ± 8.14 **
HSFD+Ibrolipim	7.92 ± 1.45 ^##^	9.00 ± 2.83 ^##^	0.10 ± 0.01 ^##^	47.55 ± 5.38 ^##^

Values are means ± SD, n = 5 in each group; * *P *< 0.05, ** *P *< 0.01 *vs*. CD group; ## *P *< 0.01 *vs*. HSFD group.

**Figure 1:**
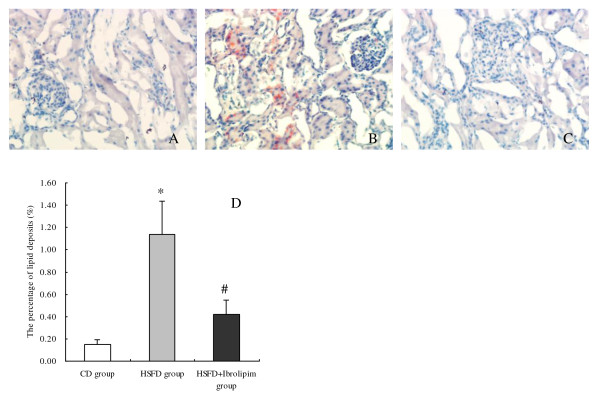
**Representative photomicrographs of Oil Red O staining in frozen kidney sections from Chinese Bama minipigs**. No apparent lipid droplets, appeared as red spots, were observed in the control group (1A) and the HSFD+Ibrolipim group (1C). There were marked deposition in tubular epithelial cells and small amounts in glomeruli of Oil Red O-stainable lipid in the HSFD group (1B) (× 200 magnification). (1D) The percentage of Oil Red O-positive area was used for comparison (mean ± SD). * *P *< 0.01 *vs*. CD group; # *P *< 0.01 *vs*. HSFD group.

Oil Red O staining of kidney sections showed minimal or absent lipid deposits in the kidneys of the CD group (Figure 1A; the percentage of positive area: 0.15% ± 0.04%) and the HSFD+Ibrolipim group (Figure 1C; 0.42% ± 0.13%, *P *< 0.001 *vs*. HSFD group). On the other hand, marked deposition in tubular epithelial cells and small amounts in glomeruli of Oil Red O-stainable lipid were observed in HSFD group (Figure 1B; 1.14% ± 0.30%, *P *< 0.001 *vs*. CD group). Corresponding to biochemical analysis of lipid composition, these results strongly indicated that there were excessive amounts of lipid accumulation in the kidneys of HSFD pigs, and the accumulation could be eliminated by treating with Ibrolipim.

### Effects of Ibrolipim on plasma and renal LPL activity in minipigs

Postprandial postheparin plasma LPL activity significantly increased in the HSFD group compared with the CD group, and even more in the HSFD+Ibrolipim group than the HSFD group (Table 3).

Renal tissue LPL activity decreased 28.57% in the HSFD group compared with the CD group, and increased 100% after the treatment with Ibrolipim compared with the HSFD group (Table 3).

### Effects of Ibrolipim on renal LPL mRNA and protein expression in minipigs

**Figure 2:**
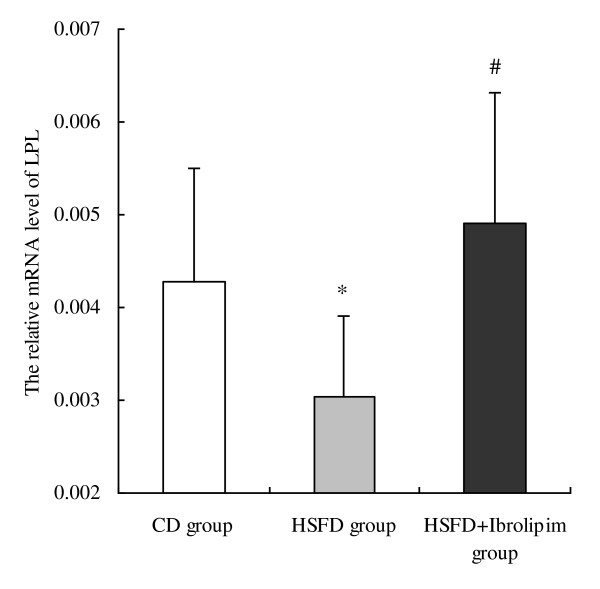
**The mRNA expression of lipoprotein lipase in the kidneys of Chinese Bama minipigs performed by real-time PCR**. The relative mRNA levels, normalized to an internal control β-actin, were calculated according to the formula 2^-ΔCT^. Data are the means ± SD, * *P *< 0.01 *vs*. CD group; # *P *< 0.01 *vs*. HSFD group.

Quantitative real-time PCR analysis showed that LPL mRNA levels decreased 28.81% in the kidney tissues of the HSFD group compared with the CD group. In contrast, it increased 61.51% in the HSFD+Ibrolipim group compared with the HSFD group (Figure 2).

**Figure 3:**
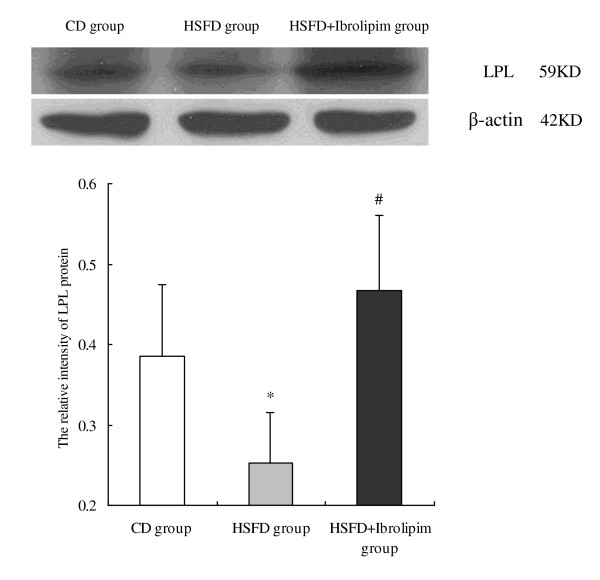
**Western blot and densitometric quantification of lipoprotein lipase protein in the kidneys of Chinese Bama minipigs**. The expression intensity of LPL protein relative to that of β-actin was calculated for comparison. Data are the means ± SD, * *P *< 0.01 *vs*. CD group; # *P *< 0.01 *vs*. HSFD group.

Western blot analysis showed that the protein expression of LPL was decreased in the kidneys of HSFD *vs*. CD minipigs, and HSFD-induced down-regulation of LPL was increased by the administration of Ibrolipim (Figure 3).

**Figure 4:**
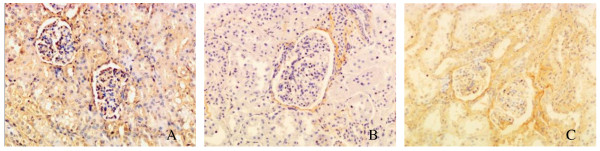
**Representative photomicrographs of immunohistochemical staining for lipoprotein lipase protein in kidney sections from Chinese Bama minipigs**. The positive staining appeared as brown in the glomerular and tubulointerstitial cells of the control group (4A), the HSFD group (4B) and the HSFD+Ibrolipim group (4C). Original magnification × 200.

Immunohistochemical analysis showed that HSFD decreased the immunoreactivity of LPL in the glomerular endothelial, parietal epithelial, and tubular epithelial cells compared with the CD group. Ibrolipim reversed the decrease compared with the HSFD group (Figure 4).

It was worthy to note that the reduction in LPL protein expression was accompanied by a parallel reduction in LPL mRNA of renal tissues for the HSFD group. In contrast, Ibrolipim stimulated the diet-induced down-regulation of LPL in the kidney.

### Effects of Ibrolipim on renal morphology in minipigs

**Figure 5:**
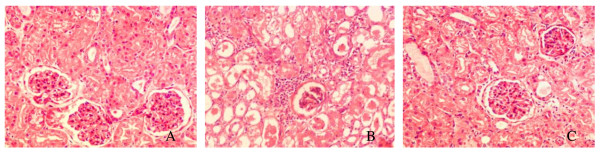
**Representative photomicrographs of HE staining in kidney sections from Chinese Bama minipigs**. (5A) Control group; (5B) HSFD group; (5C) HSFD+Ibrolipim group. Original magnification × 200.

HE staining clearly revealed glomerular hypertrophy, mesangial expansion, parietal layer of Bowman's capsule thickening, inflammatory cell infiltration, and dilated tubules within hyaline casts in the HSFD-fed minipigs (Figure 5B) compared with the control minipigs (Figure 5A). These abnormalities associated with HSFD feeding were attenuated by Ibrolipim treatment (Figure 5C).

### Correlations between contents of triglyceride and cholesterol and LPL activity in kidneys

**Figure 6:**
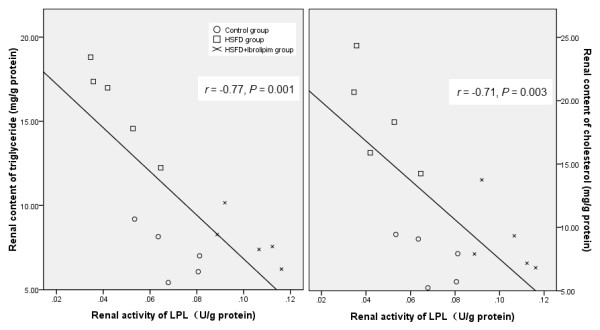
**Correlations between contents of triglyceride and cholesterol and LPL activity in kidney**.

As shown in Figure 6, triglyceride content and cholesterol content were inversely correlated with LPL activity in kidneys (*r *= -0.77, *P *= 0.001; *r *= -0.71, *P *= 0.003, respectively).

## Discussion

In our research, HSFD-feeding to minipigs for 5 months induced weight gain, hyperglycemia, hyperinsulinemia, insulin resistance, hypertriglyceridemia, hypercholesterolemia and microalbuminuria. The diet induced excessive amounts of lipid deposits in the kidney, as shown by increased Oil Red O staining and significantly higher renal contents of triglyceride and cholesterol. We also found that HSFD resulted in renal morphological abnormality, which was characterized by glomerular hypertrophy, mesangial expansion, inflammatory cell infiltration and dilated tubules within hyaline casts. Results from our study do confirm those previous findings and support the theory that diet-induced lipid metabolism disorder and excessive renal lipid accumulation are contributing to the development of DN [3,6,8,29].

Furthermore, our study has the following novel findings. The first is that the elevation of renal triglyceride and cholesterol contents is correlated with a significant reduction in renal LPL activity (Figure 6). This is accompanied by a parallel reduction in mRNA and protein expression of renal LPL. The specific immunolocalization with anti-LPL antibodies by using immunohistochemistry is expressed in the glomerular endothelial, parietal epithelial, and tubular epithelial cells, where ectopic lipid deposition is observed in HSFD-fed minipigs with Oil Red O staining. However, there are conflicting reports that LPL is expressed by different types of renal cells, and its mRNA, mass and activity in kidneys vary widely among different species, such as human, mink, mouse, rat and Chinese hamster [11,12,30-33]. Mesangial cells, but not epithelial cells, express *LPL *mRNA in human and rat [11]. Immunostaining for kidney LPL indicates that the enzyme is present in tubular epithelial cells of mouse and mink [12] and vascular endothelium of glomeruli in guinea pig [34]. To our knowledge, the present study is the first to report that the expression and activity of LPL are evaluated in the kidney of Chinese Bama minipigs, and down-regulated by HSFD for 5 months.

The second novel finding of our study is that HSFD feeding increases postprandial postheparin plasma LPL activity while decreases the mRNA and protein expression and activity of renal LPL. However, the mechanism for this phenomenon is not clear. Previous study has shown that plasma LPL activity increases and reaches a maximum at 6 h after intake of the oral fat load in humans [35]. Moreover, LPL activity is regulated according to nutritional state in a tissue-specific manner [10]. Adipose tissue LPL activity rises rapidly after feeding by a post-transcriptional mechanism in guinea pigs and mice [36,37]. In SD rats, a high-fat diet differentially affects LPL activity in muscle and adipose tissue, reducing the former and greatly increasing the latter, which tends to induce fat storage and insulin resistance [22]. It is, therefore, speculated that HFSD seems to promote the release of LPL from peripheral tissues such as adipose tissue to blood.

Several studies have revealed that peripheral catabolism of triglyceride-laden lipoproteins (i.e., CM and VLDL) is impaired and postheparin LPL activity and mass are reduced in clinical nephrosis [15,38,39]. In animal models of kidney disease, significant reduction of LPL activity, mRNA level or protein mass is observed in the cardiac muscle, skeletal muscle and adipose tissues [16-18,33,40,41]. These studies are consistent with an important role for acquired LPL deficiency in the pathogenesis of dyslipidemia and renal diseases. In sharp contrast, studies *in vitro *have shown that LPL enhances the binding of VLDL to mesangial cells *via *a heparan sulfate-dependent mechanism [13], increases cellular triglyceride accumulation *via *enzymolysis [14], and stimulates both the proliferation of these cells and the expression of PDGF [13]. In baby hamster kidney (BHK) and human embryonal kidney 293 (HEK 293) cells, the stimulating effect of LPL on HDL_3 _selective cholesteryl ester uptake is independent of lipolysis [42]. Above all, both clinical and experimental studies are still producing conflicting findings that LPL may have pro-nephrogenic or anti-nephrogenic effects.

Our minipigs with diet-induced DN exhibits a significant LPL deficiency in kidney tissue *per se*, and this seems to imply that renal LPL have beneficial effects on lipid metabolism and renal protection. Therefore, LPL activator might protect the kidney from injury to lipid accumulation. In fact, losartan improves lipid metabolism abnormality and increases LPL activity in adipose tissue of rats with renal artery stenosis [43]. Oral administration of 100 mg/kg Glycyrrhizic acid up-regulates the LPL mRNA expression in the kidney of high-fat diet induced obese rats [33].

The third novel finding of the present study is that LPL selective activator Ibrolipim, given for 5 months in combination with HSFD, significantly reduces hyperglycemia, hyperinsulinemia, insulin resistance, hypertriglyceridemia, microalbuminuria, renal fat accumulation, and improves pathological injury, all consistent with the increase in renal LPL activity and expression. These existing evidences suggest that the renoprotective effects of Ibrolipim on suppressing triglyceride and cholesterol accumulation in kidney may be directly attributable to the activation of the down-regulation of renal LPL mRNA and protein and activity induced by HSFD.

Of course, additional mechanisms may be responsible for the effects of Ibrolipim. Firstly, Ibrolipim reduces ectopic lipid deposition in the heart, skeletal muscle, liver and pancreas in diet-induced diabetic animal model [22,23] through increasing LPL activity in soleus skeletal muscle and myocardium with an increase in fat oxidation [22]. Therefore, it is possible that the increase of renal LPL expression and activity can also lead to an increase of triglyceride utilization in kidney. Secondly, Ibrolipim has been proved to be highly effective in increasing plasma HDL-C through mediating reverse cholesterol transport from peripheral tissues to the liver to protect against the excessive accumulation of cholesterol [44]. Thirdly, Ibrolipim exhibits the significant inhibitory activity of *in vitro *mesangial cell proliferation [45]. Further studies are warranted to determine the mechanisms of Ibrolipim in the suppression of renal lipid accumulation and renal disease.

## Conclusions

In summary, renal LPL plays a crucial role in the pathophysiology of lipid metabolism and the pathogenesis of nephropathy, and activation of renal LPL is renoprotective in diet-induced DN. However, the fact that our and others' studies are still producing conflicting findings with respect to the role of LPL only suggests that our understanding of LPL and Ibrolipim are not thorough. It is undeniable that Ibrolipim, a lipoprotein lipase agonist, has been proved to have potential benefit for prevention and treatment of DN.

## List of abbreviations

CD: control diet; CM: chylomicrons; Cr: creatinine; DN: diabetic nephropathy; HDL-C: high-density lipoprotein cholesterol; HOMA: homeostatic model assessment; HSFD: high-sucrose and high-fat diet; LPL: lipoprotein lipase; mALB: microalbumin; PDGF: platelet-derived growth factor; TC: total cholesterol; TG: triglyceride; VLDL: very-low-density lipoproteins.

## Competing interests

The authors declare that they have no competing interests.

## Authors' contributions

YL and ZBW contributed to design, animal experiment, analysis and writing of the manuscript; WDY to design and planning of the study; QKL, MBC, JY, HGL, CZ and XHZ to animal experiment, data collection and analysis. All authors read and approved the final manuscript.
